# Vascular remodeling and TSLP/angiogenin overexpression in severe mixed asthma

**DOI:** 10.1186/s12931-025-03133-9

**Published:** 2025-02-28

**Authors:** Francesca Bertolini, Vitina M.A. Carriero, Elisa Arrigo, Giuseppe Guida, Stefano Levra, Stefano Pizzimenti, Mirella Profita, Isabella Gnemmi, Antonino Di Stefano, Fabio L.M. Ricciardolo

**Affiliations:** 1 Department of Clinical and Biological Sciences, University of Turin, Turin, TO Italy; 2 Rare Lung Disease and Respiratory Pathophysiology Unit, Severe Asthma, San Luigi Gonzaga University Hospital, Orbassano, Turin, TO Italy; 3 Institute of Translational Pharmacology, section of Palermo, National Research Council (IFT-CNR), Palermo, 90146 PA Italy; 4 Divisione di Pneumologia and Laboratorio di Citoimmunopatologia dell’Apparato Cardio Respiratorio, IRCCS, Respiratory Rehabilitation Unit of Gattico-Veruno, Istituti Clinici Scientifici Maugeri, Novara, 28013 NO Italy

**Keywords:** Matrix-related remodeling, Vascular remodeling, Mixed/neutrophilic asthma, Severe asthma, Asthma exacerbation

## Abstract

**Background:**

Asthma with neutrophilic/mixed inflammation is a difficult-to-control clinical phenotype. Currently, vascular and matrix airway remodeling in asthma with neutrophilic/mixed inflammation is not well known. We aimed to evaluate the differences in vascular/smooth muscle/matrix related asthma remodeling in eosinophilic (EOS) and mixed/neutrophilic (MIXED) bronchial phenotypes in relation to asthma severity and exacerbation frequency.

**Methods:**

In this cross-sectional study, α-SMA^+^ cells (100µM beneath the basement membrane [BM]), BM thickness, vascular remodeling-related biomarkers (angiogenin, vascular endothelial growth factor [VEGF], CD31 and Protease-activated receptor 2 [PAR2]), alarmins (TSLP and Interleukin (IL)-33) were evaluated in bronchial sections from 40 mild-to-severe asthmatics (EOS: *N* = 19 and mixed/neutrophilic: *N* = 19/2) and 7 control subjects (CTRL).

**Results:**

The number of CD31^+^ and angiogenin^+^ cells was higher in MIXED than in EOS asthmatics (*p* < 0.05). In severe MIXED CD31^+^, TSLP^+^, α-SMA^+^, and angiogenin^+^ cells increased compared to mild MIXED/EOS or severe EOS (*p* < 0.05), but BM thickness was higher in severe vs. mild EOS (*p* < 0.05). MIXED frequent exacerbators had higher numbers of CD31^+^ and TSLP^+^ cells, whereas MIXED non-exacerbators had increased PAR2^+^ cells. CD31^+^ cells correlated with impairment of pulmonary functions, number of exacerbations, ICS dose, bronchial neutrophils, angiogenin, α-SMA, TSLP and IL-33 (*p* < 0.05). Finally, CD31 > 97.17 cells/mm^2^, angiogenin > 35.36 cells/mm^2^, and functional parameters such as FEV_1_, FEV_1_/FVC, TLC and FRC (%pred.) were found to be predictors of severe MIXED asthma.

**Conclusion:**

The severe or frequent exacerbator asthmatics with bronchial mixed inflammatory profile are characterized by increased number of vessels and overexpression of TSLP and angiogenin, suggesting a pathogenetic link between mixed eosinophilic and neutrophilic inflammation and vascular remodeling.

## Background

Asthma is a heterogeneous disease, characterized by chronic airway inflammation and variable airflow obstruction associated with airway remodeling [1]. Currently, asthma is stratified into clinical and inflammatory phenotypes: the latter are classified as T2-high and T2-low phenotypes based on allergic sensitization, F_E_NO levels, and blood eosinophils. In addition, at the laboratory level, the inflammatory cell assessment by induced sputum identifies eosinophilic, neutrophilic, mixed, and paucigranulocytic inflammatory phenotypes [2]. Recent evidence unveils that exposure to environmental stimuli, infections, and airborne factors during the lifespan leads to an overlap of these inflammatory phenotypes highlighting the heterogeneous and complex nature of asthma^2^. The neutrophilic/mixed asthma phenotype represents the most severe clinical phenotype and still little is known about its pathophysiological mechanisms [3, 4]. Neutrophils provide the first line of defense in response against several pathogens that kill through, for instance, the neutrophil extracellular traps (NETs), but they are also required for tissue repair and the development and maintenance of a healthy vasculature [5].

Airway remodeling consists of changes in structural components of the airway walls such as mucosal fibroblast activation, reticular basal membrane (BM) thickening, neo-angiogenesis and smooth muscle hypertrophy/hyperplasia [6]. Vascular remodeling is characterized by angiogenesis and vasodilation, resulting in airway wall thickening and airflow reduction. Angiogenesis involves vascular endothelial growth factor (VEGF) and angiogenin, which induce vascular endothelial cell proliferation, migration, and tubule formation [1, 7]. Previous evidence shows that these molecules are increased in older asthmatics and are associated with asthma severity [2, 8, 9]. A study performed on bronchial biopsies from asthmatics showed an increased number of vessels and percentage of vessel area compared to controls, highlighting the critical role that vascular remodeling plays in the onset, progression and asthma severity [10]. Recent evidence highlights the important role of human neutrophils in production and release of proangiogenic factors. Different in vivo and in vitro studies revealed that NETs promptly contribute to angiogenesis underlining the important role that neutrophils might play in the processes of angiogenesis and vascular remodeling [4, 11]. It is well known that airway smooth muscle cells and fibroblasts drive cellular and structural changes in the airway remodeling processes, but the possible involvement of neutrophils in tissue remodeling is beginning to be recognized [12].

Several studies have reported an association between inflammatory mediators related to neutrophils such as IL-6, IL-8, IL-17 and TNF-α and airway remodeling, although the underlying mechanisms are not fully understood [13]. Other findings support the involvement of neutrophils in inflammation and airway remodeling in allergic asthma [14], although less is known about the remodeling features associated with bronchial neutrophilia in asthma.

Several molecules associated with inflammation process in asthma may also play a role in asthma vascular remodeling [10]. Protease-activated receptor 2 (PAR2) plays a critical role in inflammatory processes, inducing increased vascular permeability, leukocyte rolling, adhesion and extravasation [15]. Furthermore, PAR2 activation induces MMP-9 release from airway epithelial cells, suggesting a possible role in vascular airway remodeling [16, 17]. Recent evidence also supports the involvement of the epithelial cytokines such as thymic stromal lymphopoietin (TSLP) and interleukin (IL)-33, upstream cytokines known as bronchial epithelial cell-derived alarmins, in asthma airway remodeling through the activation of different immune and or structural cells [18–19]. Specifically, in a murine asthma model study, IL-33 induced angiogenesis and vascular permeability [20], while TSLP contributed to the release of VEGF-A from human lung macrophage [21].

Based on these observations, we hypothesized that specifically cytokines play a critical role in vascular remodeling mechanism in patients characterized by bronchial neutrophilic/mixed compared to eosinophilic phenotype. With this study, we aimed to evaluate the differences in vascular/smooth muscle/matrix-related remodeling biomarkers expression comparing asthmatics with isolated eosinophilic vs. mixed/isolated neutrophilic phenotypes. We also investigated whether the above-cited biomarkers are associated with asthma severity and frequency of exacerbations.

## Methods

### Patients

For this observational cross-sectional study, forty bronchial biopsies obtained from asthmatic outpatients referred to the Severe Asthma, Rare Lung Disease and Respiratory Pathophysiology Unit of the San Luigi Gonzaga University Hospital (Orbassano, Torino, Italy) were selected. Asthma and severe asthma were defined according to GINA [22] and ERS/ATS Guidelines [23]. Written informed consent was obtained from each subject. The study conformed to the Declaration of Helsinki and was approved by the local Ethics Committees (San Luigi Gonzaga University Hospital: protocols 1759/2008 and 14871/2009). Clinical data and patient history were obtained at enrollment when spirometry, post-bronchodilation reversibility tests and hematological exams were performed. Based on the GINA 2017 [24] definition we ruled out patients with asthma-COPD overlap (ACO). As a control, we included 7 bronchial biopsies from subjects without a diagnosis of asthma and/or COPD who underwent bronchoscopy for other clinical reasons (e.g., unknown origin of hemoptysis).

At the time of the study, all patients were in stable condition and none had been treated with theophylline, antibiotics, antioxidants, mucolytics, and/or systemic glucocorticoids in the six weeks prior to bronchial biopsy.

**Fig. 1:**
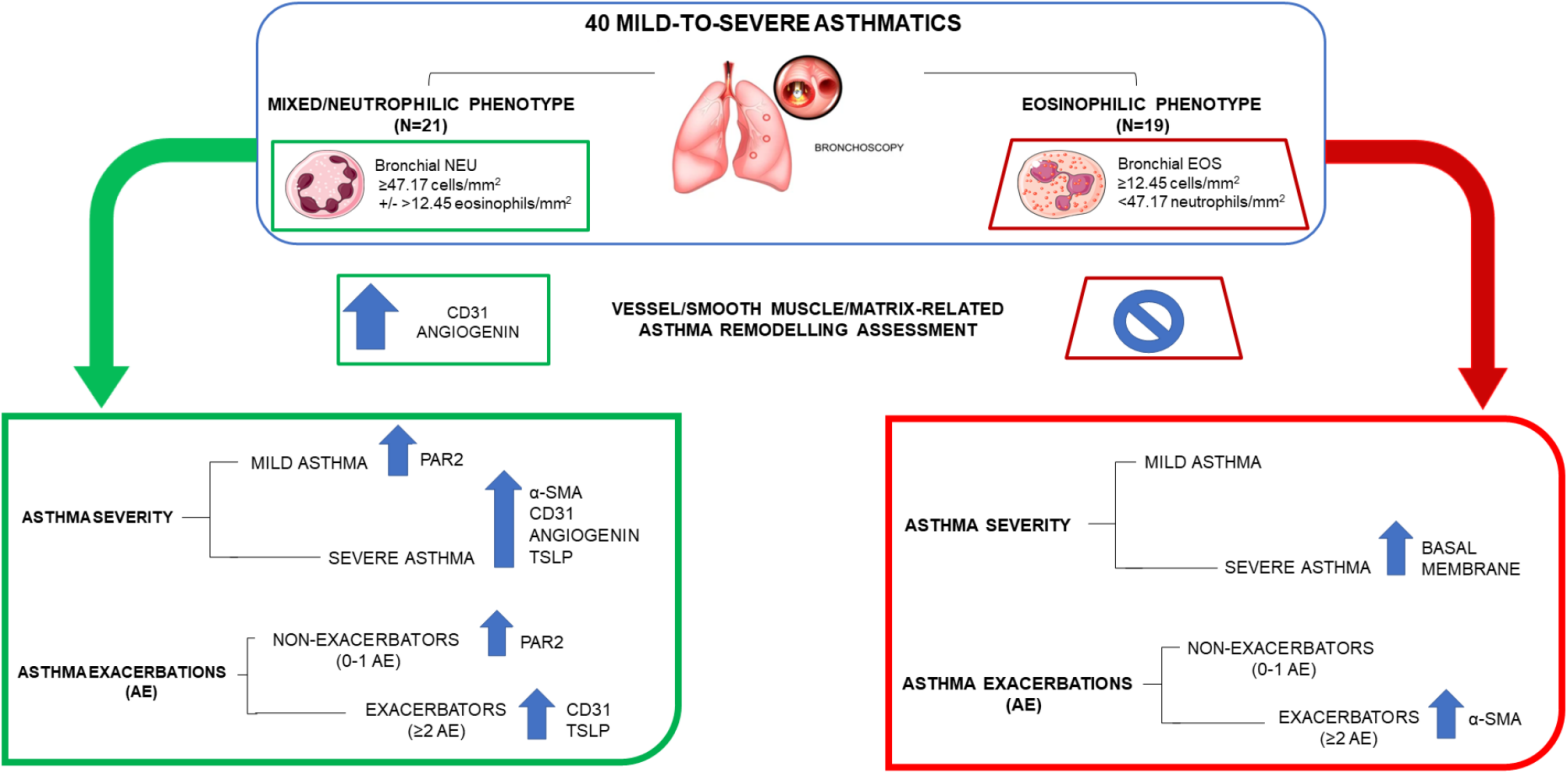
Study design and summary of the main findings obtained from immunohistochemistry staining on bronchial biopsies of asthmatic patients included in the study. The blue upward arrow indicates an increase than the comparative group (mixed/neutrophilic vs. eosinophilic, mild vs. severe, exacerbators vs. non-exacerbators); the “not allowed” symbol indicates no differences

Asthmatics were divided into severe (SA: patients requiring fluticasone propionate ≥ 500mcg equivalence [GINA steps 4/5]), and mild (MA: patients treated with fluticasone propionate dose ranging from 125 to 375mcg equivalence)^23^ and further in exacerbator (EXA: asthmatics who reported ≥ 2 asthma exacerbations requiring OCS burst in the previous year), and non-exacerbator (NON-EXA: exacerbation rate between 0 and 1) [25]. The schematic design of the study is reported in the Fig. 1.

### Phenotype definitions

We evaluated bronchial biopsies obtained during a previous study [26, 27]; based on neutrophils and eosinophils density in their bronchial mucosa, asthmatic patients were divided into 2 groups: isolated eosinophilic (≥ 12.45 eosinophils/mm^2^ and < 47.17 neutrophils/mm^2^, *N* = 19) and mixed/neutrophilic (≥ 47.17 neutrophils/mm^2^, *N* = 21, Fig. 1) as previously described [26]. In the mixed/neutrophilic groups two patients were characterized by high bronchial neutrophils without significant number of bronchial eosinophils (≥ 47.17 neutrophils/mm^2^ and < 12.45 eosinophils/mm^2^) and 19 patients were characterized by high levels of bronchial neutrophils with significant number of bronchial eosinophils (≥ 47.17 neutrophils/mm^2^ and ≥ 12.45 eosinophils/mm^2^).

### Evaluation of clinical, functional and blood parameters

Pulmonary function test was performed by assessing spirometry and lung volumes before and 15 min after the administration of albuterol (400 µg) using body plethysmograph (Vmax Encore 62, Carefusion, Germany). Post-bronchodilation (PB) FEV_1_ (%pred.) < 80% and FEV_1_/FVC ratio ≤ 0.70 were used as thresholds to describe the presence of patients with fixed airflow obstruction (FAO) [28]. Fractional exhaled nitric oxide (F_E_NO) at the flow of 50 mL/s by means of a chemiluminescence analyzer (Eco Medics CLD88 sp, Duernten, Switzerland) was measured as previously described [26]. White blood cell (WBC) count and WBC differential count were performed based on optical and impedance characteristics using a Cell-Dyn Sapphire (Abbott, Rome, Italy) automated hematology analyzer (data were expressed as an absolute number). Fibrinogen plasma content was turbidimetrically measured using ACL-TOP CTS 700 (Werfen Italia, Milan, Italy) coagulation analyzer [29]. Smoking history was defined as ≥ 10 pack-years, based on previous data. The patients who had stopped smoking at least one year before were considered former smokers. Skin prick tests and specific serum total IgE levels identified allergic patients according to validated criteria [30], and patients sensitized to 2 or more allergens were considered as poly-sensitized [31].

### Fiberoptic bronchoscopy collection and immunohistochemistry

As previously described, bronchoscopy was performed in all subjects using a flexible fiberoptic bronchoscope (Pentax FB-35 18P; Asahi Optical Co. LTD, Tokyo, Japan) and bronchial biopsy samples were collected [26]. Immunostaining was performed on 6 μm-thick frozen sections (CTRL and asthmatic subjects) using specific antibodies against eosinophil cationic protein (ECP, 1:100, rabbit polyclonal IgG, cod. bs-8615R, Bioss, Woburn, MA, USA) and neutrophil elastase (1:100, mouse monoclonal IgG, clone NP57, Agilent Dako, Santa Clara, CA, USA); CD4 for T helper lymphocytes (1:100 mouse monoclonal, clone 4B12, Dako, Denmark); tryptase for mast cells (1:500 mouse monoclonal, clone G3, EMD Millipore Corporation, CA, USA); CD68 for macrophages (1:100 mouse monoclonal Ab-3, clone KP1, Thermo Fisher Scientific, UK); CD31 for the number of vessels (1:50 mouse monoclonal, JC/70A, Thermo Fisher Scientific, UK); mouse anti-human α-SMA (1:100 DAKO cytomation, Milan, Italy); VEGF (1:200, rabbit polyclonal ab46154, Abcam, Cambridge, UK); angiogenin (1:200, rabbit polyclonal, ab189207, Abcam, Cambridge, UK); PAR2 (1:200, rabbit monoclonal, ab180953, Abcam, Cambridge, UK); TSLP (1:000 rabbit polyclonal, ABT330, Merck, Milan, Italy), IL-33 (1:200 mouse monoclonal antibody, sc-517600, Santacruz, Texas, USA).

Appropriated biotinylated secondary antibodies (Vector Laboratories, Peterborough, UK) were then applied (1:200); antibody binding was revealed by the use of Bloxall Blocking Solution (SP-6000, Vector Laboratories) and colour development was achieved by treatment with DAB substrate (Sigma Aldrich S.r.l., Milan, Italy).

Cell counts were performed on immunohistochemically stained tissue at 40x magnification by the same operator blinded to subject identification and diagnosis and when it possible a minimum of 8 high power fields per section have been analyzed. Immunostained cells were quantified in the area 100 μm below the basement membrane (lamina propria) in multiple non-overlapping high-power fields until the entire available area was covered. The number of positive cells/mm^2^ was calculated as the average of all the cellular counts performed in all fields. We quantified the immunostained cells in which at least a portion of the nucleus seen close to immunopositivity. Capillaries were identified as structures positively stained with a primary anti-CD31 antibody with a semicircular to circular shape [9]. Basal membrane thickness was measured in Hematoxylin-Eosin stained cryostat slices of bronchial biopsies using the method described in previous study [32, 33].

### Statistical analysis

Statistical evaluations were performed with GraphPad Prism 9.0 (GraphPad Software, San Diego, CA, USA). Data distributions were assessed using the D’Agostino-Pearson test. Unpaired T-test (Welch-corrected, if different standard deviations were detected) or Mann-Whitney test were used to compare differences between groups. Chi-squared (χ2) tests were used to compare frequencies. Optimal cut-off values of severe neutrophilic asthma (clinical and immunohistochemical parameters) were determined by receiver operating characteristic (ROC) curves; the area under the ROC curve (AUC) was used to measure the accuracy of each score. Odds ratios (OR) and 95% confidence intervals (CI) were used to estimate the predictors of severe neutrophilic asthma. Outliers were identified using ROUT methods and excluded from analyses [34]. Correlations were determined using the Pearson or Spearman correlation coefficient. *P*-values equal to and < 0.05 were considered significant [35].

## Results

### Evaluation of clinical, functional, biological characteristics

Characteristics of 47 subjects included in the study are reported in Table 1. The 40 asthmatic subjects included 19 eosinophilic (EOS) and 21 mixed/neutrophilic (MIXED), while the control group was composed of 7 subjects.

**Table 1:** Clinical, functional, and biologic parameters of patients

	CTRL (*n* = 7)	All asthmatics (*N* = 40)	Eosinophilic (*n* = 19)	Mixed (*n* = 21)
Mild/Severe asthma	n.a.	22/18	12/7	10/11
Age (years)	48.6 ± 19.7 (19–74)	50.4 ± 11.3 (31–70)	50.0 ± 9.2 (34–66)	50.7 ± 13.3 (31–70)
Sex (M/F)	4/3	17/19	11/8	9/12
BMI (Kg/m^2^)	25.2 ± 3.4	26.4 ± 4.9	26.2 ± 4.7	26.2 ± 5.3
Asthma onset (mean ± SD)	n.a.	27.3 ± 16.8	31.3 ± 16.9	22.5 ± 16.1
Asthma onset (< 18 years)	n.a.	14/40 (35.0%)	5/19 (26.3%)	9/21 (42.9%)
Asthma duration (years)	n.a.	23.2 ± 17.8	18.7 ± 15.0	28.2 ± 18.7
Current smokers§	1/7 (14.3%)	2/40 (5.0%)	1/19 (5.3%)	1/21 (4.8%)
Past smokers§	0/7 (0.0%)	11/40 (27.5%)	7/19 (36.8%)	4/21 (19.0%)
Atopy	0/7 (0.0%)	23/40 (57.5%)##	9/19 (47.4%)	14/21 (66.7%)##
Polysensitized	0/7 (0.0%)	18/23 (78.2%)	8/9 (88.9%)	10/14 (71.4%)
Perennial allergen sensitization	0/7 (0.0%)	14/18 (77.7%)	5/8 (62.5%)	9/10 (90.0%)
IgE (KUI/L)	n.a.	198.1 ± 253.3	180.2 ± 250.2	196.6 ± 252.1
FeNO (ppb)¥	n.a.	31.5 ± 25.1	35.7 ± 21.1	29.3 ± 26.9
WBC (cell/µL)	6.6 ± 4.5	7.0 ± 1.7	7.1 ± 1.8	6.9 ± 1.6
Blood eosinophils (cell/µL)	57.0 ± 20.4	288.8 ± 203.5##	297.9 ± 184.4####	293.3 ± 236.5###
Blood neutrophils (cell/µL)	4613 ± 3967	3548 ± 1183	3679 ± 1347	3440 ± 1024
Blood lymphocytes (cell/µL)	1907 ± 1276	2293 ± 690.1	2236 ± 825.1	2438 ± 653.4
Blood monocytes (cell/µL)	597.0 ± 727.2	515.5 ± 183.9	540.0 ± 179.1	509.0 ± 191.1
Blood basophils (cell/µL)	13.3 ± 5.8	49.5 ± 28.0	47.9 ± 25.9	49.5 ± 32.0
pre FVC (% pred.)	116.0 ± 16.1	104.9 ± 19.0	105.2 ± 16.8	102.8 ± 20.7
PB FVC (% pred.)	n.a.	106.3 ± 15.4	109.1 ± 16.9	104.2 ± 14.2
pre FEV_1_ (% pred.)	104.4 ± 33.8	82.7 ± 24.7	87.0 ± 21.0	76.3 ± 26.9#
PB FEV_1_ (% pred.)	n.a.	86.0 ± 26.6	88.8 ± 31.0	82.7 ± 22.3
PB FEV_1_ (% pred.) (< 80%)°	n.a.	14/40 (35.0%)	5/19 (26.3%)	9/21 (42.8%)
pre FEV_1_/FVC	0.78 ± 0.0	0.65 ± 0.15	0.68 ± 0.13	0.61 ± 0.15
PB FEV_1_/FVC	n.a.	0.68 ± 0.14	0.71 ± 0.13	0.66 ± 0.15
PB FEV_1_/FVC (≤ 0,70)°	n.a.	19/40 (47.5%)	9/19 (47.4%)	10/21 (47.6%)
RV (% pred.)	n.a.	124.5 ± 44.7	117.3 ± 25.5	132.9 ± 54.4
TLC (%pred.)	n.a.	112.5 ± 16.4	104.6 ± 10.9	119.4 ± 17.6*
FRC (%pred.)	n.a.	109.1 ± 33.5	97.4 ± 19.4	119.8 ± 39.3*
ICS/day (µg fluticasone HFA eq.)	n.a.	462.5 ± 349.4	394.7 ± 318.5	583.3 ± 422.7
OCS (≥ 6 months/year)	n.a.	4/40 (10.0%)	0/19 (0.0%)	4/21 (19.0%)
Mean exacerbation rate	n.a.	1.5 ± 1.7	1.1 ± 0.9	1.9 ± 2.1
Frequent exacerbation (≥ 2/year)	n.a.	14/36 (35.0%)	5/19 (26.3%)	9/21 (42.9%)

Continuous variables are presented as mean ± SD. For the age variable, the min to max range is also provided. Incidences are reported as occurrence/exposed cases and as percentage

Exacerbation means severe exacerbation with the use of OCS burst

n.a. = not applicable

The symbol “§” indicates the inclusion of patients with smoking history of ≥ 10 Pack/Year

The symbol “¥” indicates that we excluded current smokers F_E_NO values

The symbol “°” indicates the number of subjects with fixed airway obstruction

*: *p* < 0.05 compared to eosinophilic, ##: *p* < 0.01, ####: *p* < 0.001 vs. CTRL

The CTRL, EOS, and MIXED groups were comparable in age, sex, BMI and smoking history.

The number of atopic patients was increased in all asthmatics and MIXED compared to CTRL (*p* < 0.01), while the MIXED and EOS did not differ in serum IgE levels and allergen sensitization pattern.

Functional residual capacity (FRC) and total lung capacity (TLC) levels were the only lung function parameters higher in MIXED groups compared with EOS (*p* < 0.05); for what concerns the other pulmonary variables studied, no differences were revealed. All 40 asthmatics, EOS and MIXED groups showed higher blood eosinophil concentrations in comparison to CTRL (*p* < 0.01); for other blood count cells no differences were observed among the groups.

### Inflammatory cell counts in the bronchial mucosa

Table 2 shows the comparisons between the inflammatory cell counts in the bronchial mucosa of study subjects. In MIXED we found higher numbers of bronchial eosinophils (*p* < 0.05), neutrophils (*p* < 0.001), and CD4^+^ cells (*p* < 0.01) than CTRL and, in addition, an increased number of neutrophils compared to the EOS group (*p* < 0.001). Moreover, we observed significant increases in bronchial eosinophil (*p* < 0.05), neutrophil (*p* < 0.05), and CD4^+^ (*p* < 0.05) cell counts in EOS compared to CTRL. Finally, we observed higher numbers of bronchial eosinophils (*p* < 0.05), neutrophils (*p* < 0.01), and CD4^+^ cells (*p* < 0.05) in all 40 asthmatics than CTRL. As reported in Table 2, in the bronchial lamina propria CTRL subjects are characterized by 6.8 ± 5.5 bronchial eosinophils/mm^2^, 8.4 ± 3.3 bronchial neutrophils/mm^2^, 4.2 ± 4.1 bronchial T helper lymphocytes/mm^2^, 137.2 ± 85.2 bronchial macrophages /mm^2^ and 36.7 ± 29.2 bronchial mast cells/mm^2^ highlighting less inflammatory degree compared with asthmatic groups.

**Table 2:** Inflammatory positive cells in the bronchial mucosa of patients

Cell counts mm^2^ lamina propria	CTRL (*N* = 7)	All asthmatics (*N* = 40)	Eosinophilic (*n* = 19)	Mixed (*n* = 21)
ECP^+^ (Bronchial eosinophils)	6.8 ± 5.5	35.1 ± 19.1^#^	41.1 ± 28.5^#^	37.7 ± 21.9^#^
NE^+^ (Bronchial neutrophils)	8.4 ± 3.3	56.2 ± 38.2^##^	23.3 ± 11.1^#^	91.1 ± 36.6****/^####^
CD4^+^ (Bronchial T helper lymphocytes)	4.2 ± 4.1	27.6 ± 17.9^#^	25.6 ± 20.1^#^	29.7 ± 16.0^##^
CD68^+^ (Bronchial macrophages)	137.2 ± 85.2	241.5 ± 177.5	281.8 ± 174.2	207.9 ± 178.0
Mast cells/tryptase+	36.7 ± 29.2	89.1 ± 111.6	78.0 ± 60.6	103.6 ± 152.5

Continuous variables are presented as mean ± SD

***: *p* < 0.001 compared to Eosinophilic; ^#^: *p* < 0,05, ^##^: *p* < 0,01; ^####^: *p* < 0,001 vs. CTRL

### Remodeling markers and alarmins expression

**Fig. 2:**
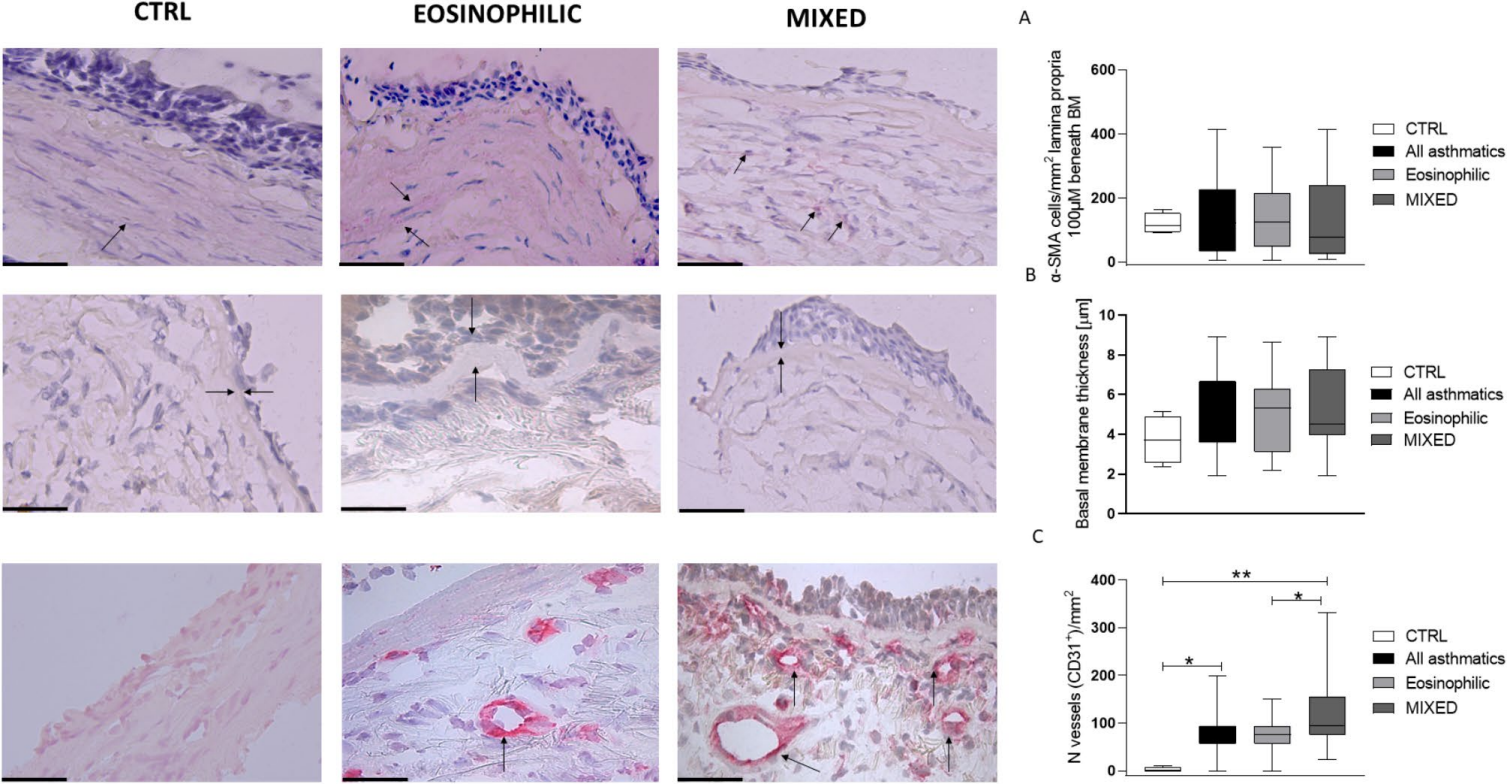
Representative photomicrographs showing cells staining positively for the markers studied in bronchial biopsies of control (left), eosinophilic asthma (middle), and mixed/neutrophilic (MIXED) asthma (right) subjects (40× magnification). Arrows indicate positively stained cells. **Panel A** shows the data of α-SMA 100 μm, **panel B** shows the basal membrane and **panel C** shows the number of vessels (CD31^+^ cells). *: *p* < 0.05; **: *p* < 0.01. CTRL = control. Data are presented as box plots (25-75th percentiles), and bars (10-90th percentiles) and the horizontal line indicates the mean value in the respective group. The differences between the groups are assessed by Unpaired *T* test or Mann-Whitney test

**Fig. 3:**
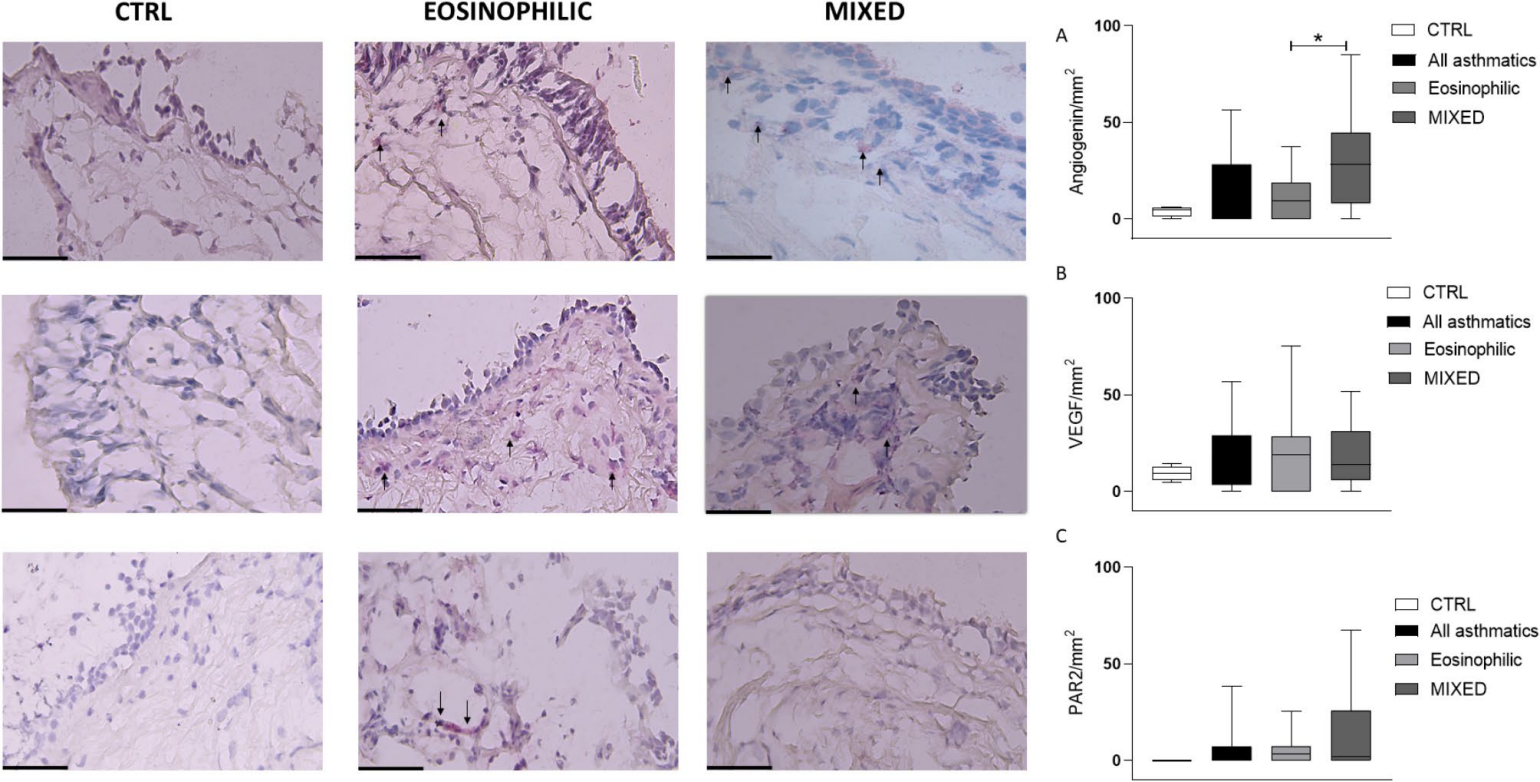
Representative photomicrographs showing cells staining positively for the markers studied in bronchial biopsies of control (left), EOS asthma (middle), and mixed/neutrophilic (MIXED) asthma (right) subjects (40× magnification). Arrows indicate positively stained cells. **Panel A** shows the data of angiogenin, **panel B** shows the VEGF^+^ cells and **panel C** shows PAR2^+^ cells. **: *p* < 0.01. CTRL = control. Data are presented as box plots (25-75th percentiles), and bars (10-90th percentiles) and the horizontal line indicates the mean value in the respective group. The differences between the groups are assessed by Unpaired *T* test or Mann-Whitney test

**Fig. 4:**
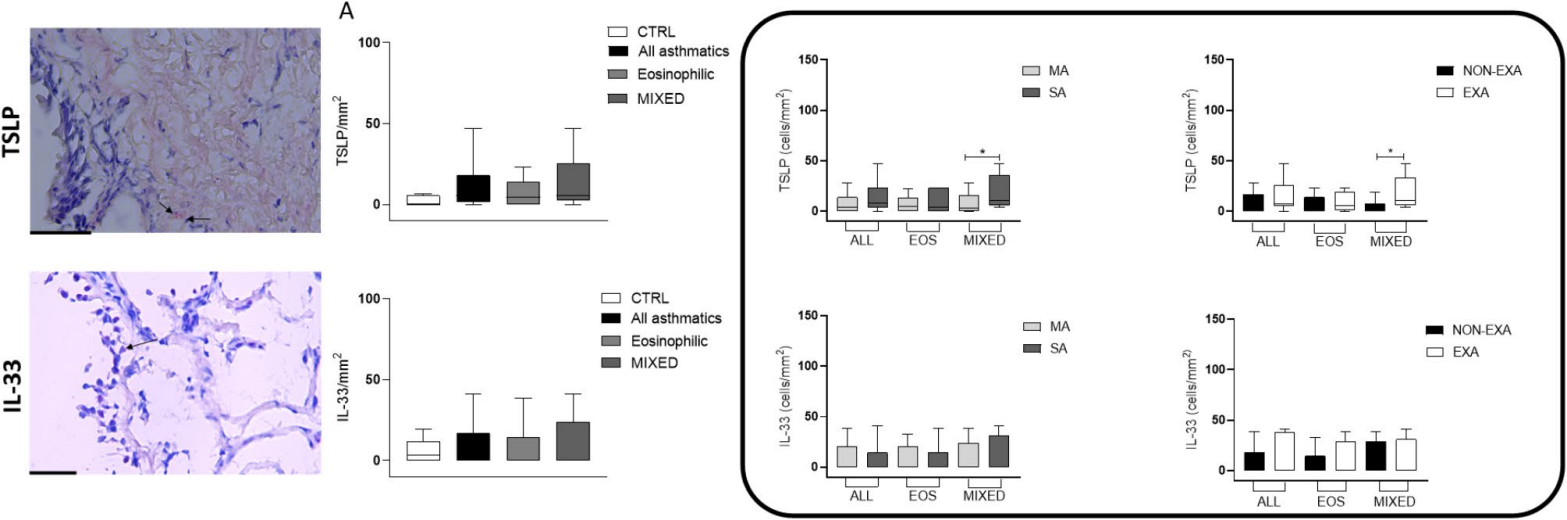
Representative photomicrographs showing cells staining positively for TSLP and IL-33 in bronchial biopsies (40× magnification, arrows indicate positively stained cells). **Panel A** reported data of TSLP and IL-33 in the bronchial mucosa of control subjects and asthmatic patients stratified into EOS and MIXED groups. The box groups the subanalysis in mild (MA) and severe (SA) and in exacerbators (EXA) and non-exacerbators (NON-EXA). EOS = eosinophilic, MIXED = mixed/neutrophilic. Data are presented as box plots (25-75th percentiles), and bars (10-90th percentiles) and the horizontal line indicates the mean value in the respective group. The differences between the groups are assessed by Unpaired *T* test or Mann-Whitney test

Immunohistochemistry assessing the remodeling biomarkers revealed higher number of vessels (CD31^+^ cells) in the MIXED group versus EOS and CTRL groups (*p* < 0.05 and *p* < 0.01 respectively, Fig. 2), while angiogenin^+^ cells were more numerous in MIXED compared to EOS and CTRL without reaching statistical significance (*p* < 0.05 and *p* = 0.06 respectively, Fig. 3). Furthermore, all asthmatics showed a higher CD31^+^ cells in the lamina propria than the CTRL group (*p* < 0.05, Fig. 2). The expressions of α-SMA 100 μm, VEGF, PAR, TSLP, and IL-33 along with BM thickness were similar among the groups (Figs. 2, 3 and 4).

In a subsequent analysis, the patients of each inflammatory group (MIXED and EOS) were stratified into MA vs. SA and EXA vs. NON-EXA.

**Fig. 5:**
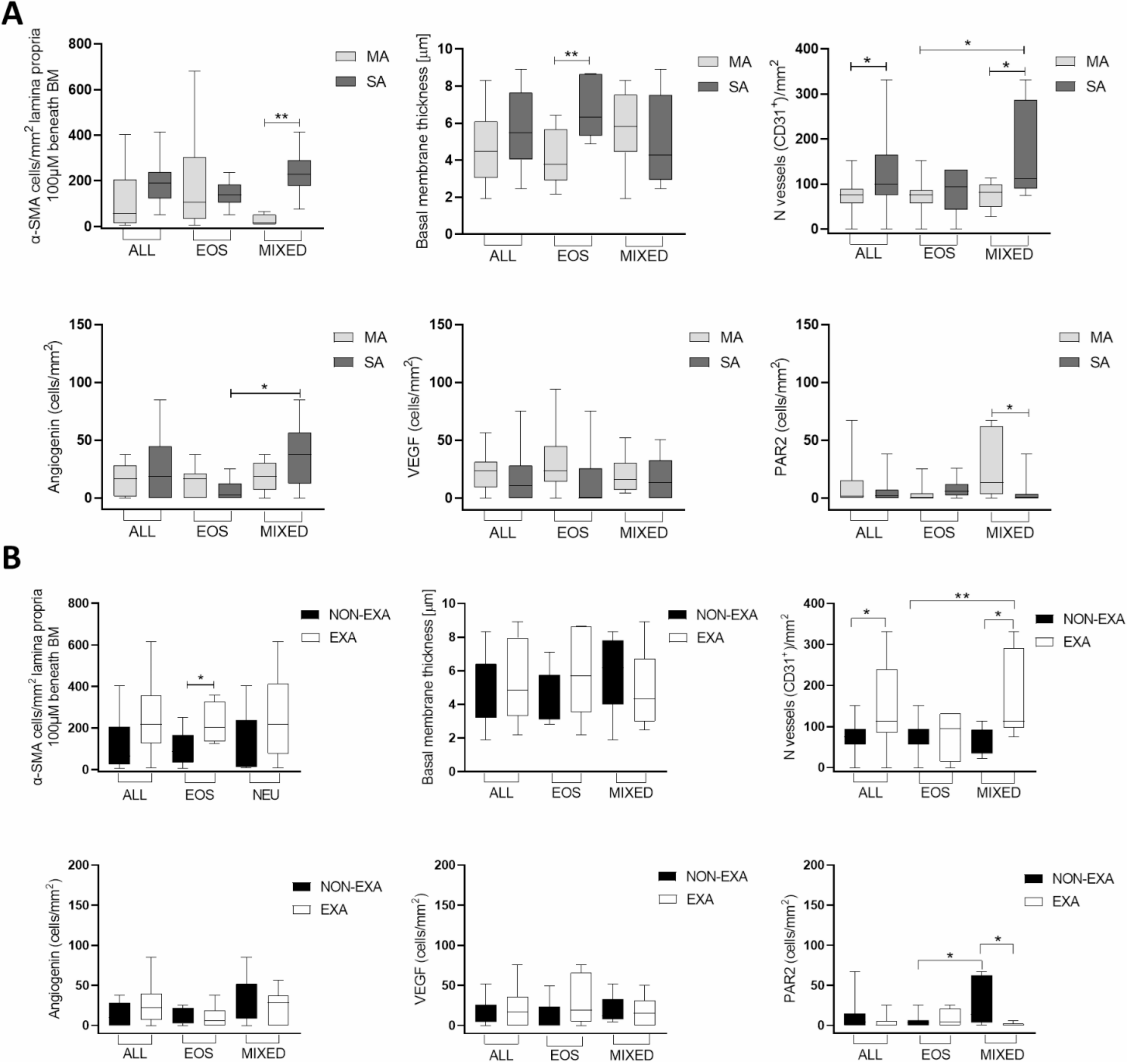
(**A**) Box plots of the remodeling biomarkers in mild (MA) and severe (SA) asthmatics. (**B**) Box plots of the remodeling biomarkers representing the group division into exacerbators (EXA) and non-exacerbators (NON-EXA). ALL = all asthmatics, EOS = eosinophilic, MIXED = mixed/neutrophilic. Data are presented as box plots (25-75th percentiles), and bars (10-90th percentiles) and the horizontal line indicates the mean value in the respective group. The differences between the groups are assessed by Unpaired *T* test or Mann-Whitney test

As showed in Fig. 5 MIXED SA had higher expression of α-SMA 100 μm compared to MIXED MA (*p* < 0.01), CD31 versus MIXED MA and EOS MA (*p* < 0.05), angiogenin versus EOS SA (*p* < 0.05), and TSLP versus MIXED MA (*p* < 0.05, Fig. 4).

MIXED MA had higher expression of PAR2 in the lamina propria than MIXED SA (*p* < 0.05, Fig. 5). BM was thicker in the EOS SA (*p* < 0.01) compared to the EOS MA. All severe asthmatics independently of inflammatory phenotype had a higher number of vessels than all MA (*p* < 0.05, Fig. 5). VEGF and IL-33 expression resulted not influenced by severity grade (Figs. 4 and 5).

Furthermore, we observed that the number of vessels increased in MIXED EXA compared to both MIXED and EOS NON-EXA (*p* < 0.05), and in all EXA group than all NON-EXA (*p* < 0.05, Fig. 5). Interestingly, PAR2^+^ cells were enriched in MIXED NON-EXA group than MIXED EXA (*p* < 0.01) and EOS NON-EXA (*p* < 0.05, Fig. 5). EOS EXA showed more elevated α-SMA 100 μm compared to EOS NON-EXA (*p* < 0.05, Fig. 5), and TSLP^+^ cells were increased in MIXED EXA than NON-EXA (*p* < 0.05, Fig. 4).

No differences among groups were observed for IL-33, BM, angiogenin and VEGF markers (Figs. 4 and 5).

### Correlations analyses

**Fig. 6:**
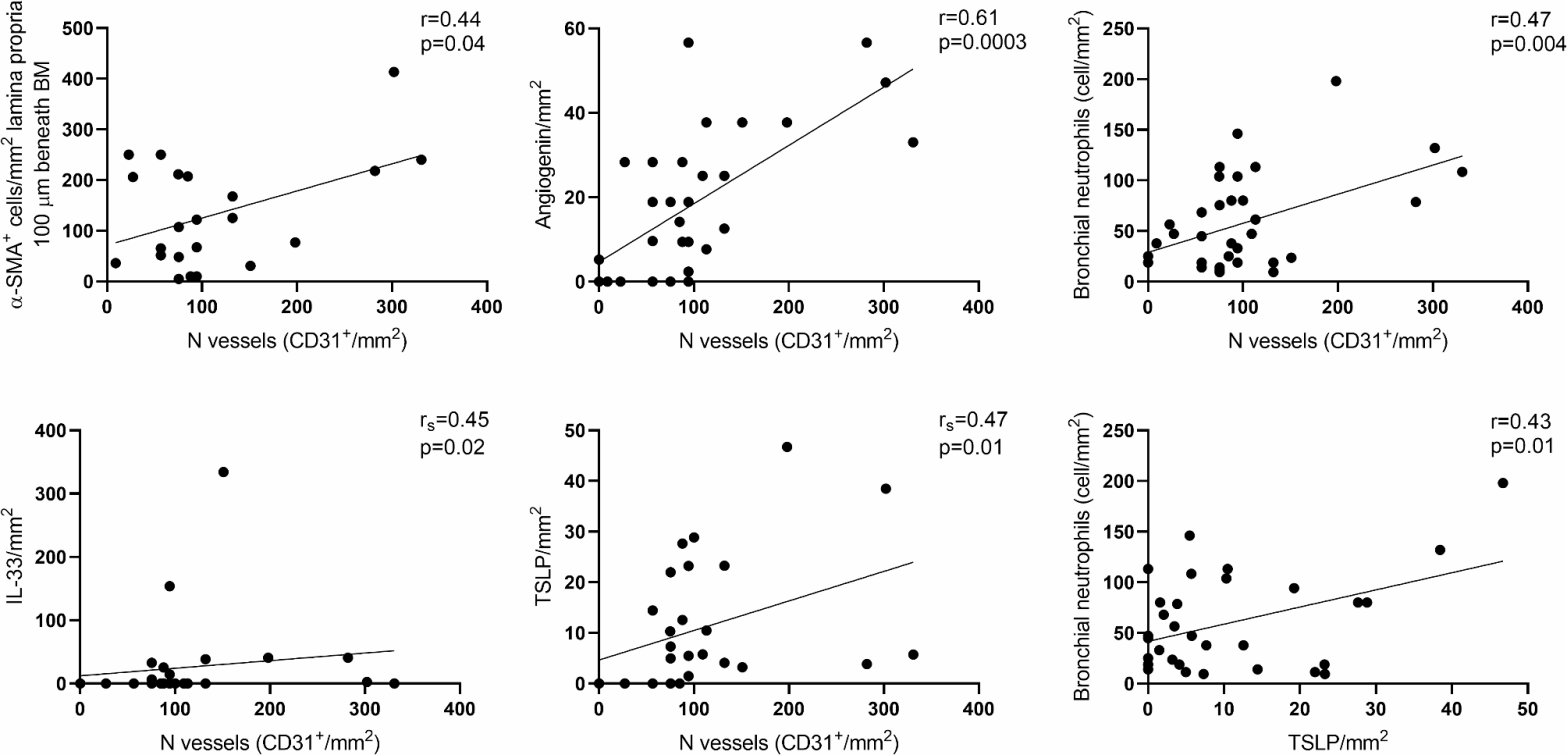
Correlations between the number of vessels, alarmins, smooth muscle/matrix-related airway remodeling and bronchial neutrophils in all asthmatic patients. Straight-line represents the best-fitting line. r = Pearson correlation coefficient, r_s=_ Spearman correlation coefficient

Analyzing the relationship between matrix/smooth muscle/vascular-related airway remodeling markers, inflammatory cells, and alarmins, we found the following correlation: the number of vessels positively correlated with α-SMA 100 μm (*r* = 0.44; *p* = 0.04), bronchial neutrophils (*r* = 0.47, *p* = 0.004), angiogenin^+^ cells (*r* = 0.61, *p* = 0.0003) and IL-33 (r_s_=0.45, *p* = 0.02). The number of TSLP^+^ cells positively correlated with CD31^+^ cells (r_s_=0.47, *p* = 0.01) and bronchial neutrophils (*r* = 0.43, *p* = 0.01). The correlation analyses are shown in Fig. 6.

**Fig. 7:**
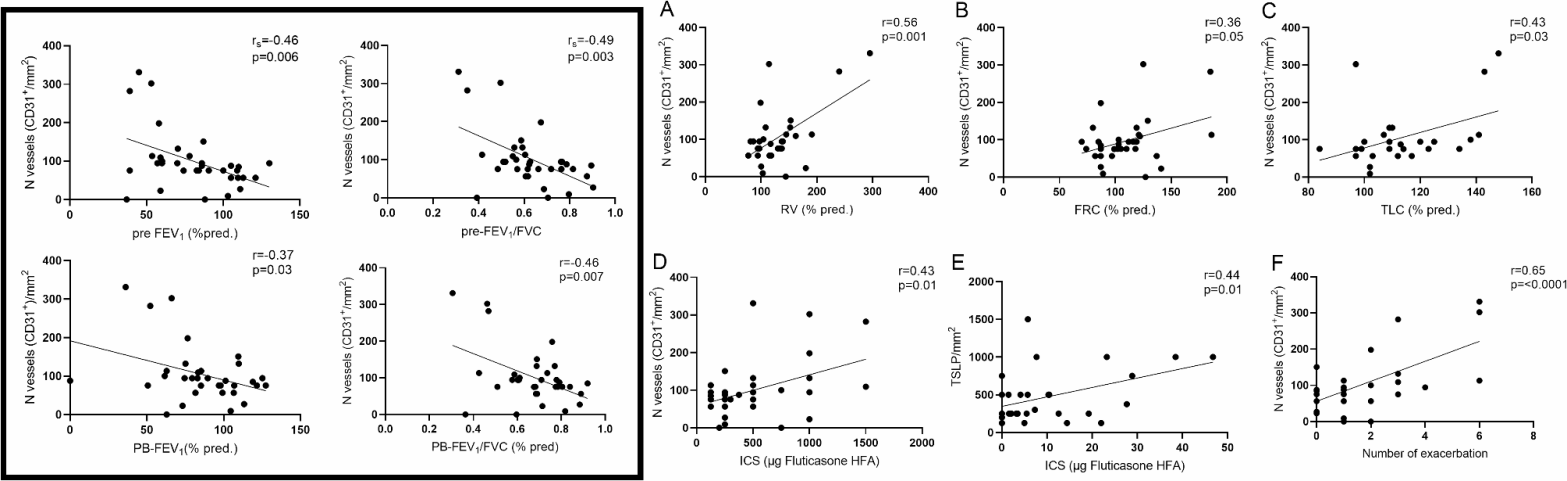
Correlations between the number of vessels and pulmonary function (**Panel A-C**) in all asthmatic population. The box groups all the correlations pertaining FEV_1_ and FEV_1_/FVC pre- and post-bronchodilators (PB) with CD31^+^ cells. Panel D-E show positive correlations between number of vessels or TSLP^+^ cells and ICS dose and Panel F shows the positive correlation between CD31^+^ cells and the number of exacerbations. Straight-line represents the best-fitting line. r = Pearson correlation coefficient, r_s=_ Spearman correlation coefficient

We found a negative correlation between the number of vessels and pre- and post-bronchodilator (PB) (% pred.) FEV_1_ (r_s_=-0.46, *p* = 0.006; *r*=-0.37, *p* = 0.03) and FEV_1_/FVC (r_s_=-0.49, *p* = 0.003; *r*=-0.46, *p* = 0.007) and positive correlation with RV (*r* = 0.56, *p* = 0.001), TLC (*r* = 0.43, *p* = 0.03), and FRC (*r* = 0.36, *p* = 0.05). The number of vessels, moreover positively correlated with both number of exacerbation (*r* = 0.65, *p* < 0.0001) and dose of ICS (*r* = 0.43, *p* = 0.01), while TSLP positively correlated with ICS dose (*r* = 0.44, *p* = 0.01). The summary of these analyses is reported in Fig. 7.

**Fig. 8:**
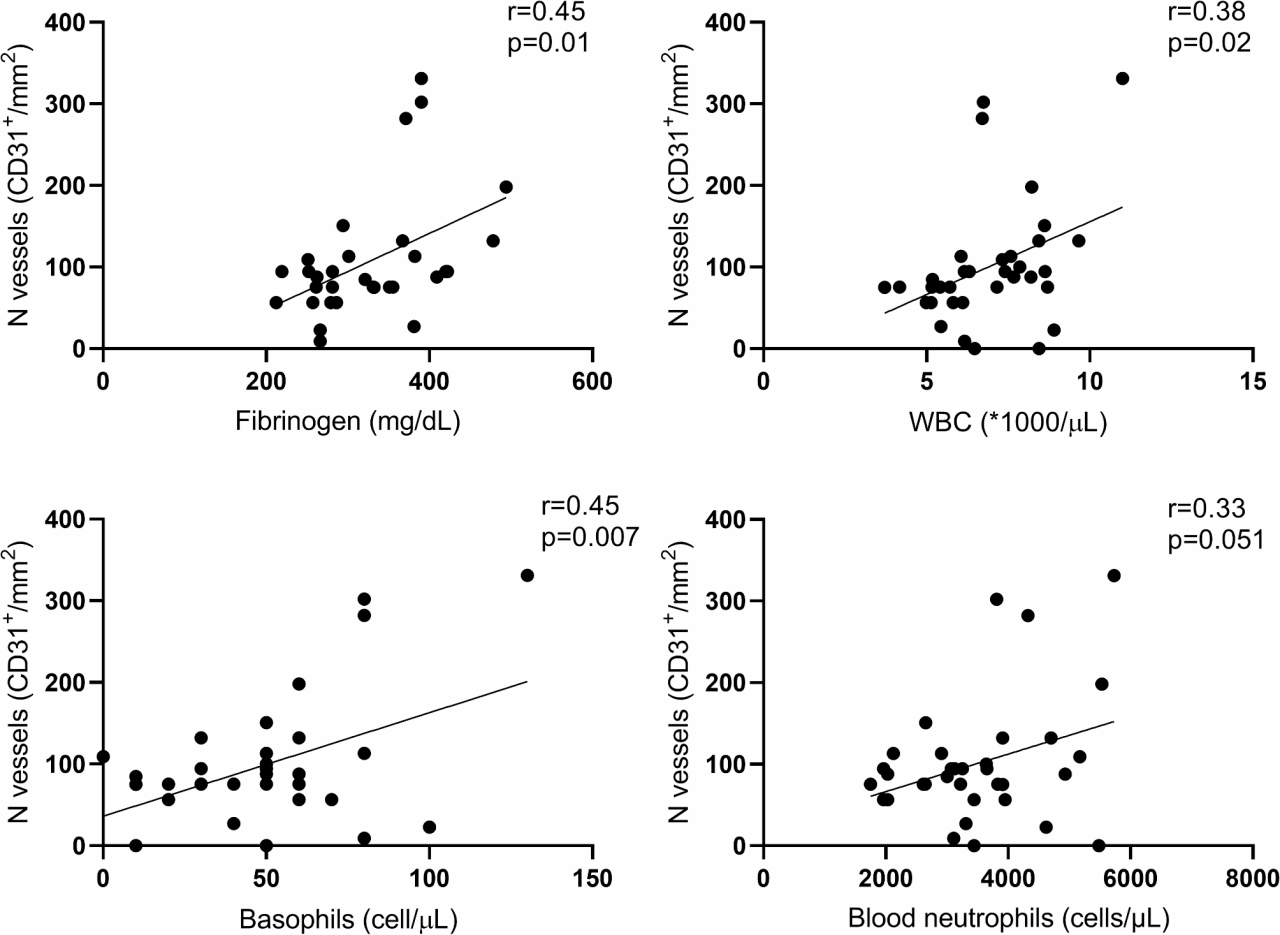
Correlations between the number of vessels and blood markers (fibrinogen, WBC, basophils and neutrophils). Straight-line represents the best-fitting line. r = Pearson correlation coefficient

Finally, CD31^+^ cells positively correlated with blood parameters as fibrinogen (*r* = 0.45, *p* = 0.01), WBC count (*r* = 0.38, *p* = 0.02), basophils (*r* = 0.45, *p* = 0.007) and blood neutrophils (*r* = 0.33, *p* = 0.05, Fig. 8).

### Predictors of severe mixed/neutrophilic asthma

**Fig. 9:**
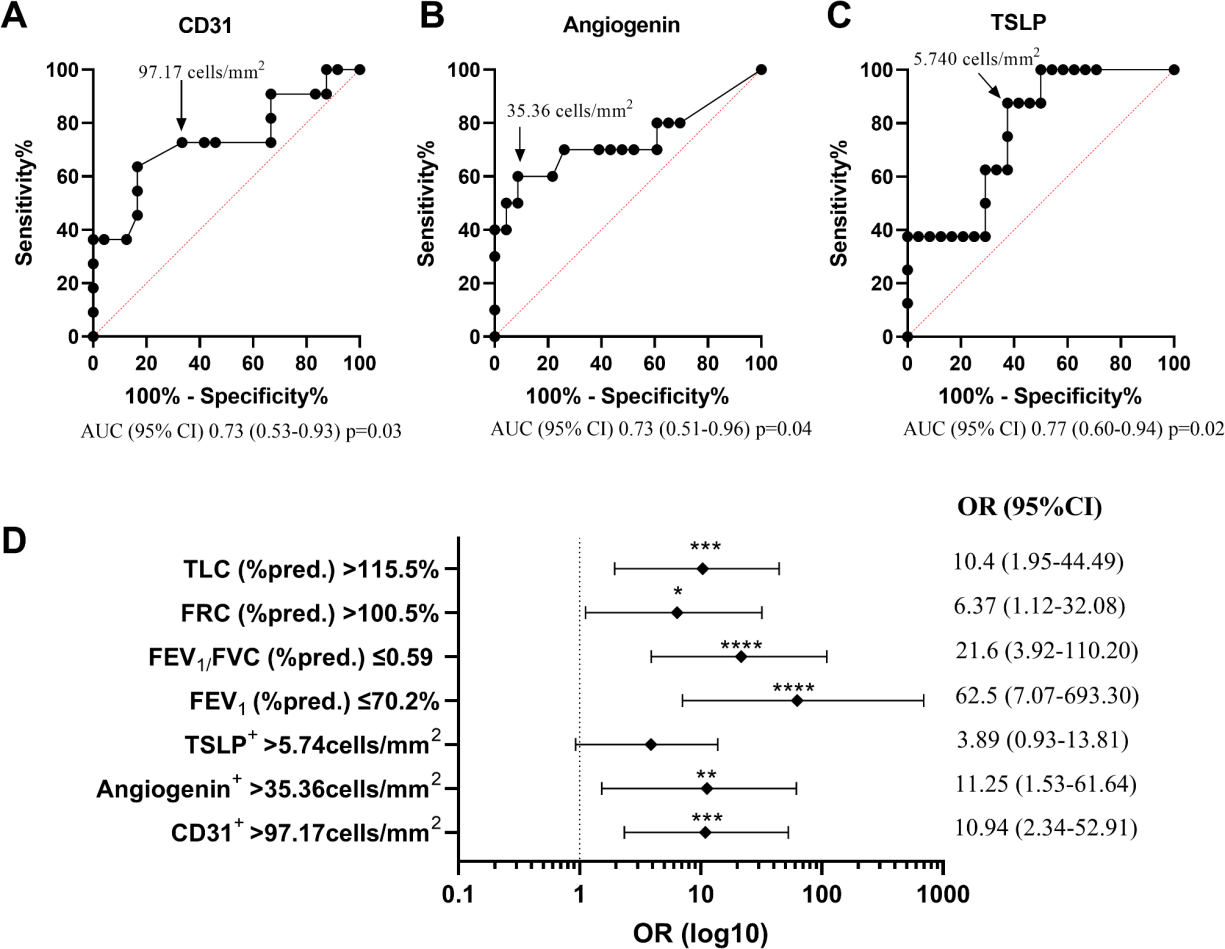
ROC curves for optimal cut-off points of CD31, Angiogenin and TSLP (**A-C**). (**D**) Forest plot indicating predictors of severe mixed/neutrophilic asthma. The points represent the confidence interval (95%). OR: odds ratio, **: *p* < 0.01; ***: *p* < 0.001

ROC curve analyses provided the best cut-off values of number of vessels, angiogenin, TSLP and lung functional parameters to discriminate severe mixed/neutrophilic asthma (Fig. 9A-C). Patients with values of CD31 > 97.17 cells/mm^2^ were 4 times more likely to be severe MIXED asthmatics (AUC 0.73; diagnostic OR = 3.8); sensitivity was 63.6%, specificity 83.3%. Patients with values of angiogenin > 35.36 cells/mm^2^ had a 6-fold higher probability of being severe MIXED asthmatics (AUC 0.73; diagnostic OR = 5.8); sensitivity was 50.0%, specificity 91.3%. Furthermore, patients with values of TSLP > 5.74 cells/mm^2^ had a 2-fold higher probability of being a severe MIXED asthma (AUC 0.77; diagnostic OR = 2.0); sensitivity was 75.0% and specificity 62.5%.

Finally, we assessed the predictive validity of the cut-offs and we revealed that CD31 > 97.17 cells/mm^2^, angiogenin > 35.36 cells/mm^2^, FEV_1_ (%pred.) ≤ 70.2%, FEV_1_/FVC (%pred.) ≤ 0.59, TLC > 115.5% and FRC > 100.5% were significant predictors of severe MIXED asthma (Fig. 9D; *p* < 0.01). TSLP > 5.74 cells/mm^2^ was not a significant predictor for identifying severe MIXED asthma based on our model.

We point out that the exclusion of the two isolated neutrophilic patients did not change the statistical significance of the overall results.

## Discussion

In the current study, we evaluated the potential impact of bronchial neutrophils in conjunction with bronchial eosinophils on vascular/smooth muscle/matrix-related airway remodeling in bronchial biopsies of control subjects and mild-to-severe asthmatics stratified based on their inflammatory phenotypes.

Regarding the clinical characteristics, we observed that compared to isolated eosinophilic groups patients with mixed/neutrophilic inflammation have higher TLC and FRC. It is noteworthy to point out that our asthmatic population as a whole despite clinically appropriate ICS and OCS treatment had a higher F_E_NO levels and a proportion of frequent asthma exacerbators. In particular, the MIXED group used higher doses of ICS and had higher rate of frequent exacerbation suggesting a potential corticosteroid resistance [36].

Evidence reported that mild and severe asthmatics had a distinct airway remodeling [33, 37], although no differences in inflammatory phenotypes has been described. In the current study some of the markers of smooth muscle/matrix-related remodeling, such as α-SMA and BM, and VEGF, PAR2, TSLP, IL-33 were not statistically significant different if compared based on the bronchial inflammatory phenotypes (MIXED vs. EOS groups). However, after dividing MIXED and EOS groups into severe and mild asthmatics, we observed that α-SMA^+^ cells at 100 μm were higher in the severe than mild MIXED, while BM was elevated in the severe versus mild EOS, proving that the smooth muscle/matrix-related markers were strongly associated with the severity of the disease [38].

Here we demonstrated, for the first time, that asthmatics characterized by mixed/neutrophilic phenotype had a higher number of vessels and angiogenin indicating that the bronchial wall of these patients had an increased neo-angiogenesis, although we cannot exclude that the higher expression of angiogenic factors in the airways was the cause and not the consequence of the mixed/neutrophilic phenotype. Our hypothesis, however, could be supported by the work of Panariti and colleagues, which showed that the IL-17 A^+^ cells in the bronchial biopsies of asthmatics were predominantly neutrophils and this cytokine in vitro stimulated the synthesis of angiogenetic factors [39].

In our sub-analyses, we also revealed that severe MIXED asthmatics had higher CD31^+^ cells than mild MIXED and EOS asthmatics. Instead, angiogenin expression was higher in severe MIXED asthmatics than in severe EOS asthmatics. The higher expression of these markers of vascular remodeling, together with the strong correlation found between the number of vessels and the number of cells positive for angiogenin and the number of bronchial neutrophils, allows us to state that in severe asthma patients vascular remodeling was supported by mixed/neutrophilic inflammation. Moreover, the association of this vascular remodeling marker with asthma severity is strengthened by its positive correlation with ICS dose. Furthermore, the number of vessels was positively correlated with plasma fibrinogen and blood neutrophils, in line with our previous findings in which plasma fibrinogen strongly correlated with blood neutrophils and exacerbation frequency [29].

These results were partially in line with the previous works of our research group, which showed that severe and elderly asthmatics patients had higher levels of CD31^+^, VEGF^+^, and angiogenin^+^ cells [1, 9]. In particular, the higher number of vessels in asthmatics with a major component of neutrophilic/eosinophilic inflammation supports the hypothesis of an immune response deviation from a classical type 2 (T2) to an alternative non-type 2 immune process in relation to additional triggers such as pathogen/environmental exposure able to induce clinical progression of the disease [2, 27]. Furthermore, the negative correlation observed between the number of vessels and both PB-FEV_1_ and PB-FEV_1_/FVC suggested that fixed airflow obstruction (FAO) could be caused by vascular remodeling. Moreover, the negative correlation between these parameters and α-SMA 100 μm underpins our previous findings in which FAO was associated with smooth muscle-related remodeling [33].

ROC analyses provided cut-off values potentially useful to identify severe mixed/neutrophilic asthmatics, based on the vascular remodeling biomarkers expression and specifically lung function parameters. Notably, the forest analysis showed that the number of vessels (> 97.17 cells/mm^2^), angiogenin (> 35.36 cells/mm^2^), pre-FEV_1_ (≤ 70.2%), pre-FEV_1_/FVC (≤ 0.59) and FRC (> 100.5%) providing a strong predictive result characterizing (OR = 10.9, OR = 11.3, OR = 62.5, OR = 21.6, OR = 10.4, respectively) severe mixed/neutrophilic asthmatics. Furthermore, also TLC (> 115.5%, OR = 6.3) appear to be a moderate predictor of mixed/neutrophilic severe asthma.

Although research on the role of the PAR2 in asthma is still ongoing, works by Palikhe and colleagues have attempted to explain the possible link of this molecule in asthma severity and exacerbations. The first study assessed the PAR2 expression on blood inflammatory cells of severe and mild-to-moderate asthmatics [40]. Severe asthmatics had higher percentages of blood monocyte expressing PAR2 than mild-moderate asthmatic patients, while no differences were observed concerning the expression of PAR2 in blood neutrophils, eosinophils and CD4 cells. A recent study [41] showed that the expression of PAR2 on blood monocytes was increased during asthma exacerbation. Conversely, we found that in our asthmatics the bronchial expression of PAR2 was higher in the mild than severe MIXED group. Furthermore, the non-exacerbator MIXED group had greater levels of PAR2 expression than MIXED exacerbators and EOS non-exacerbators. Our observation indicates that a differential regulation of PAR2 expression exists in neutrophilic asthma. The intimate mechanisms that regulate the physiological/pathophysiological role of PAR2 in neutrophilic asthmatics needs further investigation.

Instead, a potential involvement of the TSLP and IL-33 in asthma vascular remodeling emerged from interesting findings in the correlation analyses.

In our asthmatic population, we followed a positive correlation between IL-33 and the number of vessels as well as between TSLP and CD31^+^, and the number of bronchial neutrophils.

These results highlight the connection between alarmins and circulating proangiogenic progenitors required for new vessel formation as suggested in a mouse model work [42], and shed light on the significance of these alarmins, particularly of TSLP, in the mechanisms of angiogenesis regulating neutrophils airway inflammation and asthma remodeling. Furthermore, our sub-analysis revealed that TSLP^+^ cells were higher in severe and EXA MIXED groups than mild and NON-EXA MIXED, suggesting the connection of this alarmin with asthma severity, also due to the strong correlation of TSLP with ICS dose. In line with other studies [43, 44], we might speculate that TSLP plays an important role in neutrophilic airway inflammation and remodeling in exacerbators and severe asthmatics.

Recent clinical trials have demonstrated the benefits in reducing exacerbation rate after the administration of tezepelumab, anti-TSLP, and astegolimab, anti-IL-33 receptor, in severe asthmatics even with eosinophil counts < 150 or 300 cells/µL [45, 46]. Although the CASCADE trial has already shown that tezepelumab had no effect on airway remodeling (no changes in the basal membrane thickness) [47], our results may encourage the use of these novel biologics as a new therapeutic option for severe asthmatics associated with neutrophilic phenotype in an attempt to reduce vascular remodeling and the related airflow obstruction, but further investigations are needed to fully elucidate this potential benefit.

We are aware of the limitation of our study due to the cross-sectional study design, the restricted number of bronchial biopsies analyzed, and the absence of data on the protein/mRNA level of the markers studied that could have further strengthened our results. Notwithstanding, only a few specialized centers are able to reproduce our findings because of the complexity and difficulty to obtain the bronchial biopsies in asthma.

On the basis of the current study, we can conclude that in severe asthmatics and frequent exacerbators with asthma, the presence of a mixed eosinophilic and neutrophilic inflammatory pattern in the bronchial mucosa is associated with increased vascular-related remodeling and TSLP expression, although additional investigations are necessary to justify our findings.

## Data Availability

No datasets were generated or analysed during the current study.

## Abbreviations

α–SMA: α-smooth muscle actin
AUC: Area under the curve
BM: Basal membrane thickness
CTRL: Control
ECP: Eosinophil cationic protein
EOS: Eosinophilic
EXA: Exacerbators
FAO: Fixed airflow obstruction
F_E_NO: Fractional exhaled nitric oxide
FEV_1_: Forced expiratory volume in 1 s
FRC: Functional residual capacity
FVC: Forced vital capacity
ICS: Inhaled corticosteroids
IgE: Immunoglobulin E
MA: Mild asthmatics
NE: Neutrophilic elastase
MIXED: Mixed/neutrophilic
NON-EXA: Non exacerbators
OCS: Oral corticosteroid
OR: Odds Ratio
PAR2: Protease-activated receptor 2
PB: Post-bronchodilation
ROC: Receiver operating characteristic
RV: Residual volume
SA: Severe asthmatics
TLC: Total lung capacity
TSLP: Thymic stromal lymphopoietin
VEGF: Vascular endothelial growth factor
